# Fatal *Lodderomyces elongisporus* Fungemia in a Premature, Extremely Low-Birth-Weight Neonate

**DOI:** 10.3390/jof8090906

**Published:** 2022-08-26

**Authors:** Mohammad Asadzadeh, Noura Al-Sweih, Suhail Ahmad, Seema Khan, Wadha Alfouzan, Leena Joseph

**Affiliations:** 1 Department of Microbiology, Faculty of Medicine, Kuwait University, Jabriya 46300, Kuwait; 2 Microbiology Department, Maternity Hospital, Shuwaikh 70031, Kuwait

**Keywords:** *Lodderomyces elongisporus*, *Candida parapsilosis* complex, fungemia, extremely low-birth-weight neonate, neonatal intensive care unit (NICU)

## Abstract

Many rare yeasts are emerging as pathogens, causing invasive infections in susceptible hosts that are associated with poor clinical outcome. Here, we describe the first and fatal case of *Lodderomyces elongisporus* fungemia in a premature, extremely low-birth-weight neonate after spontaneous vaginal delivery. The bloodstream isolate was identified as *C. parapsilosis* by the VITEK 2 yeast identification system and as *L. elongisporus* by PCR-sequencing of the internal transcribed spacer (ITS) region of ribosomal DNA. Antifungal susceptibility testing data for the isolate, performed by the broth microdilution-based MICRONAUT-AM assay, showed susceptibility to all nine antifungal drugs tested. Despite the initiation of treatment with liposomal amphotericin B, the patient died on the same day that the blood culture yielded yeast growth. This is the first report of *L. elongisporus* bloodstream infection in a neonate as the previous nine cases reported in the literature occurred in adult patients. The crude mortality rate for invasive *L. elongisporus* infection is 50%, as only 5 of 10 patients survived.

## 1. Background

Invasive fungal infections (IFIs) are an important cause of morbidity and mortality in hospitalized patients, particularly those with compromised immunity or other debilitating critical illnesses [1,2]. Premature neonates, considered as immunocompromised subjects due to their immature immune system, are at a greater risk for IFIs as they are more likely to also undergo invasive procedures such as intubation, parenteral feeding, central venous catheterizations as well as treatment with broad-spectrum antibiotics [3,4,5,6,7,8,9,10,11,12,13]. Yeasts are now recognized as the third most prevalent cause of healthcare-associated bloodstream infections [12]. Many rare yeasts are emerging as human pathogens, causing invasive infections in premature (gestational age of ≤35 weeks), very low-birth-weight (<1000 g) neonates that are difficult to treat [1,7,10,13,14,15].

*Lodderomyces elongisporus*, an ascomycetous yeast, shares many phenotypic and physiological characteristics with the three species (viz. *Candida parapsilosis* sensu stricto, *Candida orthopsilosis*, and *Candida metapsilosis*) of the *C. parapsilosis* complex [16,17]. *L. elongisporus* forms turquoise color colonies, while *C. parapsilosis* complex members form pink or lavender colonies on chromogenic CHROMagar Candida medium [16,18]. Biochemical identification systems for yeasts such as VITEK 2 (bioMérieux) and API 20C (bioMérieux) also often misidentify them as *C. parapsilosis* as their databases do not include *L. elongisporus*. Specific identification is achieved only by PCR amplification or PCR amplification followed by DNA sequencing (PCR-sequencing) of the internal transcribed spacer (ITS) region and/or the D1/D2 domains of ribosomal (r)DNA or by the matrix-assisted laser desorption ionization time-of-flight mass spectroscopy (MALDI TOF-MS) [16,17,18,19].

Fungemia cases due to *L. elongisporus* are rare and are often related to immunosuppression or intravenous access devices [18,19,20,21]. Here, we describe the first case of *L. elongisporus* fungemia in an extremely low-birth-weight preterm neonate. The identity of the bloodstream isolate was confirmed by multiplex-PCR (mPCR) assay and PCR-sequencing of rDNA.

## 2. Case Report

An extremely low-birth-weight (ELBW, 710 g), preterm (23 weeks of gestational age), female neonate was born following spontaneous vaginal delivery. Antenatal complications included preterm labor, chorioamnionitis, and the administration of intrapartum antibiotics. The neonate was admitted to the neonatal intensive care unit (NICU) with ELBW, hyaline membrane disease and was managed medically with high-frequency oscillatory ventilation. She was cannulated with an umbilical arterial catheter and an umbilical venous catheter using a vacuum-assisted closure technique and was started on total parenteral nutrition together with empiric antibiotic treatment with ampicillin and amikacin planned for the next seven days. The septic screen performed on the day of admission showed no signs of sepsis. On Day 3, her abdominal ultrasound findings were normal, however, a cranial ultrasound indicated a tiny left choroid plexus cyst (1.8 mm). On Day 7, her follow-up ultrasound investigations revealed intraventricular bleeding with dilated lateral ventricles as well as the third ventricle. On Day 9, a complete septic workup was conducted again due to development of fever, including a blood culture. Antibiotic treatment was started with teicoplanin and amikacin empirically. However, with progressive deterioration, the antibiotic treatment was changed to tazocin and liposomal amphotericin B was also added to the regimen to cover for fungal infections. Despite the inclusion of an antifungal cover, the neonate expired on Day 9 due to sepsis. Subsequently, the blood sample retrieved on Day 9 yielded yeast growth.

Bloodstream yeast isolate (Kw754/21) was recovered from BACTEC Peds Plus/F culture vials after 3 to 4 days of incubation at 37 °C. The isolate formed typical yeast-like cream-colored colonies with a smooth surface and entire margins on Sabouraud dextrose agar and was processed as described previously [4,5]. The yeast isolate (Kw754/21) was initially identified as *C. parapsilosis* by the VITEK 2 yeast identification system (bioMérieux, Marcy l’Etoile, France). Genomic DNA was extracted from the bloodstream isolate using Chelex-100 as reported previously [17]. The isolate Kw754/21 was identified as *L. elongisporus* by the multiplex PCR, performed as described previously [17]. Briefly, PCR amplification was performed by using four different forward primers (*C. parapsilosis* sensu stricto-specific primer mCPF, 5′-TTTGCTTTGGTAGGCCTTCTA-3′; *C. orthopsilosis*-specific primer mCOF, 5′-TAAGTCAACTGATTAACTAAT-3′; *C. metapsilosis*-specific primer mCMF, 5′-AACTGCAATCCTTTTCTTTCTA-3, and *L. elongisporus*-specific primer mLEF, 5′-TACAGAATTTTGAGAATTGTG-3′) targeting specific sequences within the ITS-1 region of the rDNA, and one common reverse primer (mCPCR, 5′-AATATCTGCAATTCATATTACT-3′) targeting the 5.8S rRNA gene sequence. PCR amplification was carried out in a final volume of 50 μL containing 1× AmpliTaq DNA polymerase buffer I and 1 unit of AmpliTaq DNA polymerase (Applied Biosystems, Brachburg, NJ, USA), 10 pmol of mCPF, mCOF, mCMF, mLELF, and mCPCR primers, 2 μL of template DNA, and 100 μM of each dNTP. Initial denaturation at 95 °C for 5 min was followed by 30 cycles of 95 °C for 1 min, 52 °C for 30 s, and 72 °C for 1 min, with a final extension at 72 °C for 10 min. PCR products (10 μL) were run on 2% (*w*/*v*) agarose gels, processed as described previously [17]. The identification was confirmed by PCR-sequencing of the ITS region of rDNA, performed by using panfungal amplification and sequencing primers as described previously [22,23]. The ITS region was amplified by using ITS1 (5′-TCCGTAGGTGAACCTGCGG-3′) and ITS4 (5′-TCTTTTCCTCCGCTTATTGATATGC-3′) primers by using the PCR amplification reaction and cycling conditions described above. The amplicons were purified by using the PCR product purification kit (Qiagen, Hilden, Germany) according to the kit instructions and both strands of the amplicons were sequenced by using the BigDye terminator v3.1 cycle sequencing kit on the ABI 3130 xl Genetic Analyzer by following the manufacturer’s instructions (Applied Biosystems). The following sequencing primers were used: ITS1FS, 5′-ACCTGCGGAAGGATCATT-3; ITS2, 5′-TCGCTGCGTTCTTCATCGATGC-3′; ITS3, 5′-TCGCATCGATGAAGAACGCAGC-3′; and ITS4RS, 5′-GATATGCTTAAGTTCAGCG-3′. The DNA sequence of the entire ITS region, including ITS-1, 5.8S rRNA and ITS-2, was assembled and was subjected to Basic Local Alignment Search Tool (BLAST) analyses (https://blast.ncbi.nlm.nih.gov/Blast.cgi?PROGRAM=blastn&PAGE_TYPE=BlastSearch&LINK_LOC=blasthome, accessed on 10 July 2022) using nucleotide collection (nr/nt) option under GenBank database. The ITS region sequence of our isolate (Kw754/21) was 100% identical to the corresponding sequences from *L. elongisporus* reference strain CBS2606 (GenBank accession no. AY391845), as well as four other strains (Kw2486/06, Kw553/08, Kw3074/14 and Kw553/18 with GenBank accession no. LN864562, FM253093, LN713946 and LS482924, respectively) obtained previously from Kuwait [17,18,19]. Furthermore, the ITS region sequence of our isolate (Kw754/21) showed only one nucleotide difference (99.82% identity) with the TYPE material (*L. elongisporus* reference strain ATCC 11503, GenBank accession no. NR_111593), as well as with another *L. elongisporus* reference strain (CBS 2605, GenBank accession no. AY391848). These data established the identity of our isolate (Kw754/21) as *L. elongisporus*.

The in vitro antifungal drug susceptibility testing (AST) of the *L. elongisporus* isolate Kw754/21 together with four other previous strains (Kw2486/06, Kw553/08, Kw3074/14 and Kw553/18) from Kuwait and the reference strain (CBS2605) was performed by the colorimetric microdilution-based MICRONAUT-AM AST panel for yeasts (Merlin Diagnostica GmbH, Bornheim, Germany). The minimum inhibitory concentration (MIC) values were read after 24 h of incubation at 37 °C and the data were interpreted by following the manufacturer’s instructions and as described previously [24]. Although there are no clinical breakpoints for *L. elongisporus*, our isolate (Kw754/21) appeared susceptible to all nine antifungal drugs including four triazoles (fluconazole, voriconazole, itraconazole and posaconazole), three echinocandins (anidulafungin, micafungin and caspofungin) a polyene (amphotericin B) and a nucleotide analog (5-fluorocytosine) as the MIC values were considerably below the plasma levels normally achieved for these drugs (Table 1) [25].

## 3. Comments

Uncommon *Candida* species and other rare yeasts are increasingly being recognized as opportunistic pathogens. Rare yeasts species are generally resistant to one or more antifungal drugs [2,26]. These yeast species with reduced susceptibility to antifungal drugs exploit antifungal selection pressure during prophylactic/empiric treatment [24,26,27]. The increasing prevalence of these rare yeast species is attributable to many reasons, which include prolonged survival of critically ill ICU patients, extended use of broad-spectrum antibiotics, and the increasing use of intravascular catheters [27]. In addition, delays in identifying and treating uncommon yeast infections also contribute towards higher fatality rates associated with rare yeast infections [27,28,29]. As a result of the severe underlying conditions of the infected individuals, *Candida* infections are associated with a high crude mortality rate of 46 to 75% [30,31].

The actual incidence of invasive infections caused by *L. elongisporus* remains underreported as this species is frequently misidentified in clinical mycology laboratories as *C. parapsilosis* by phenotypic methods, such as the automated yeast identification systems like Vitek 2 or API 20C [18,19]. In the present study, we describe the first and fatal case of fungemia caused by *L. elongisporus* in a premature, extremely low-birth-weight neonate in Kuwait. The neonate deteriorated clinically rapidly after the onset of infection, likely due to prematurity and died due to signs and symptoms of sepsis despite initiation of treatment with liposomal amphotericin B soon after recognition of the signs of infection. A review of the literature has identified nine previous episodes of invasive infections due to *L. elongisporus* and all these cases occurred in adult (age range of 22 to 79 years) patients. The clinical details of these cases together with our case and outcome are presented in Table 2. The patients had several underlying conditions, indicating the debilitating nature of their illnesses before the onset of fungemia.

Five of ten patients died, despite treatment with one or more antifungal drugs, which is comparable to the crude mortality rate of *Candida* infections (Table 2). Three of five patients who succumbed to their infection were treated with echinocandin. Members of the *C. parapsilosis* complex, to which *L. elongisporus* is closely related, often show reduced in vitro susceptibility to echinocandins [17]. Current Infectious Diseases Society of America (IDSA) guidelines recommend using echinocandins to treat candidaemia caused by *C. parapsilosis* as echinocandin use does not negatively affects the outcome [34]. This is also reflected among fungemia cases due to *L. elongisporus*, and all five patients who survived the infection had also received echinocandins as the main drug or as part of their antifungal treatment regimen. This is also consistent with the AST data as all five clinical *L. elongisporus* isolates from Kuwait exhibited very low MICs for all three echinocandins and appeared susceptible to these drugs [18,19].

*L. elongisporus* was identified for the first time in 2008 as the causative agent of bloodstream infections [16]. In subsequent reports from other parts of the world, its identification in fungemia patients was reported (Table 2). Although *C. parapsilosis* isolates are common causes of fungemia in neonates, surprisingly, no previous invasive *L. elongisporus* cases in neonates have been described. This might be due to the fact that this yeast is still an uncommon pathogen in many geographical regions and/or it is misidentified as *C. parapsilosis* as it shares many phenotypic and physiological characteristics with members of the *C. parapsilosis* complex [16,17] and DNA sequencing or other molecular facilities are not readily available in many clinical mycology laboratories.

## 4. Conclusions

The first case of fungemia due to *L. elongisporus* in a premature, extremely low-birth-weight neonate is presented. Major risk factors for invasive fungal infections in our neonate included hyaline membrane disease, extremely low-birth-weight, and prior treatment with broad-spectrum antibiotics. A bloodstream yeast isolate was cultured, which was initially misidentified as *C. parapsilosis* by VITEK 2. The correct identification of the isolate as *L. elongisporus* was obtained by a multiplex PCR and PCR-sequencing of rDNA. Despite the initiation of treatment with liposomal amphotericin B, the neonate died on the same day when the fungemia began.

## Figures and Tables

**Table 1:** In vitro antifungal susceptibility testing results of reference strain and clinical *L. elongisporus* isolates from Kuwait.

Isolate No.	Source	Minimum Inhibitory Concentration (MIC) Values (µg/mL) for								
		FLU	VOR	ITR	POS	AFG	MFG	CAS	AMB	5-FC
CBS2605	Orange juice	0.25	≤0.008	≤0.03	≤0.008	0.02	0.02	0.03	0.13	≤0.06
Kw2486/06	Blood	0.25	≤0.008	≤0.03	≤0.008	0.02	0.02	0.03	0.5	≤0.06
Kw553/08	Blood	0.25	≤0.008	≤0.03	≤0.008	0.02	0.02	0.03	0.13	≤0.06
Kw3074/14	Blood	0.25	≤0.008	≤0.03	≤0.008	0.02	0.02	0.03	0.25	≤0.06
Kw553/18	Blood	≤0.002	≤0.008	≤0.03	≤0.008	0.02	0.02	0.03	0.06	≤0.06
Kw754/21	Blood	0.25	≤0.008	≤0.03	≤0.008	0.02	0.02	0.03	0.5	≤0.06

FLU, fluconazole; VOR, voriconazole; ITR, itraconazole; POS, posaconazole; AFG, anidulafungin; MFG, micafungin; CAS, caspofungin; AMB, amphotericin B; 5-FC, 5-fluorocytosine; CBS, Central Bureau for Schimmelcultures (recently renamed as Westerdijk Fungal Biodiversity Institute).

**Table 2:** List of reported cases of *L. elongisporus* fungemia infections.

Case No.	Country	Age in Years	Sex	Underlying Condition	Treatment	Outcome	References
1	Australia	30	M	Endocarditis, osteomyelitis and brain embolic lesions; intravenous drug user	CAS, AMB + 5-FC, VOR	Survived	[32]
2	Kuwait	63	M	Cardiovascular attack, vascular catheter	FLU	Survived	[18]
3	Qatar	22	M	Trauma victim	CAS, FLU	Died	[14]
4	Japan	39	M	Thoracoabdominal aortic replacement complicated with aortoesophageal fistula, catheter	MFG	Survived	[8]
5	Spain	79	M	COPD, diabetes mellitus, ESRD	CAS	Died	[29]
6	Korea	56	F	Lung cancer, receiving immunosuppressive agents, vascular catheter	NA	Died	[20]
7	Kuwait	71	F	Hypertension, ischaemic heart disease, peripheral vascular disease	CAS	Died	[19]
8	Australia	54	F	Stoma, total parenteral nutrition	AFG	Survived	[21]
9	USA	46	M	Intracerebral hemorrhage, aortic valve replacement	MFG, AMB, 5-FC	Survived	[33]
10	Kuwait	9 Days	F	Extremely low-birth weight, HMD	L-AMB	Died	This study

AFG, anidulafungin; CAS, caspofungin; COPD, chronic obstructive pulmonary disease; ESRD, end-stage renal disease; FLU, fluconazole; 5-FC, 5-fluorocytosine; HMD, hyaline membrane disease; L-AMB, liposomal amphotericin B; MFG, micafungin; VOR, voriconazole; NA, not available.

## Data Availability

All relevant data are available in the manuscript.

## Nucleotide Sequence Accession Numbers

Nucleotide sequence of ITS region of rDNA has been deposited to GenBank database under accession no. ON754241.1.
